# Fast-track cardiology clinics—going Dutch!

**DOI:** 10.1007/s12471-019-1304-8

**Published:** 2019-07-02

**Authors:** G. A. Somsen

**Affiliations:** Cardiology Centers of the Netherlands, Amsterdam, The Netherlands

In the Netherlands, 1.4 million people suffer from cardiovascular disease. This number will increase with more than 30% over the next 20 years. To ensure access to outpatient clinics at acceptable costs, the efficiency of outpatient health care warrants optimisation. Rapid-access or fast-track cardiology services not only enable dealing with the rising number of outpatients, but also meet patients’ expectations such as short waiting time, direct evaluation of the test results and prompt therapy if necessary.

Rapid-access clinics have shown to reduce hospitalisations, are more cost-effective and are viewed positively by patients and general practitioners [1]. It can be anticipated that rapid access reduces the time that a patient is unable to work and therefore positively affects reintegration and reduces the costs of social services. In addition, a diagnostic ‘one-stop shop’ (all diagnostic tests are performed during one visit) is efficient since a time-fragmented process results in unnecessary re-scheduling and repeated medical interviews.

This has been recognised by Lenderink and Balkestein, who started a Dutch fast-lane outpatient clinic in 2007. In their article, published in the current issue, they describe the long-term outcome of this initiative. Their study shows that a rapid-access and fast-track cardiology clinic for the evaluation of suspected cardiac disease, is safe, which is in keeping with other studies. High efficiency was shown since a cardiac origin of the complaints could be excluded in 50% of patients in a single visit.

The predictability of health-care demand plays a pivotal role in any efficient clinical process. As the authors have shown, the predominant reasons for referral are three complaints that account for 85% of the patients [2]. Using these data, specific care pathways can be planned before the patient visits the clinic, resulting in a more effective use of resources and a shorter time for the diagnostic evaluation. For example, the authors suggest Holter registration prior to the consultation in patients with palpitations which is an excellent example of innovative modification of the health-care process.

The close interaction between general practitioners and rapid-access clinics will help to better select patients who need a referral and will guarantee continuity of cardiac care. In such a context, the patient will be the ultimate winner, in terms of service as well as care. In the future, clinical decision support systems may help to further increase the quality and efficacy [3]. These systems can potentially advise the physician on the probability of disease according to the patient profile, signs and symptoms and the diagnostic test results. In addition, the most effective diagnostic or therapeutic strategy can be determined. Machine learning can be used to analyse outcome data in relation to patient profile and construct algorithms to advise on optimal individual diagnostic and/or therapeutic strategies. A further increase in efficiency of the clinical process can be anticipated.

In general, three essential requirements for high-quality, safe and scalable fast-track health care can be recognised: process supporting software, dedicated and well-trained staff, and an efficient set-up of the physical space. First, as most of the hospital IT systems lack process support, fast-track strategies cannot be scaled up. Software should minimise administrative tasks, ensure fast and automated uploading of diagnostic data and provide easy access to test results, which will enable health-care employees to focus on the patient and the clinical process. Second, all staff needs to be educated and trained to optimally perform each task in the diagnostic process. Standard operational procedures and guidelines are crucial for adequate training of staff. Uniformity of the process is a key factor for safety and efficiency and should be continuously monitored and corrected when needed. Last, the design of the physical space (clinic) should support the patient flow, allowing patients to advance through the clinic unimpeded.

To determine the added value of fast-track cardiology clinics, clinical outcome and patient satisfaction should be continuously monitored using Patient Reported Outcome Measures (PROMs) and patient reviews after each consultation. These data can be used to optimise process design, software and staff training.

Rapid access to the clinic can only be guaranteed if patients are immediately treated or returned to the referring physician, if possible, which is in accordance with the current health-care policy to make sure that patients receive the right care in the right place at the right time. In addition, follow-up consultations should be minimised which can be achieved by using remote monitoring systems (e-health) [4].

Implementing advanced diagnostic tools, such as computed tomography angiography and cardiac magnetic resonance imaging, in fast-track cardiology clinics is a challenge. It can be anticipated that the use of these advanced diagnostics will increase, as guidelines increasingly recommend their use [5].

The article of Lenderink and Balkestein is an important milestone in the developing field of outpatient clinics. It contributes to a solid base for the upscaling and optimisation of these clinics which will enable affordable, accessible and scalable health care now and in the future.
